# PD08 - Measuring direct and indirect airway hyperresponsiveness in young children with obstructive symptoms

**DOI:** 10.1186/2045-7022-4-S1-P8

**Published:** 2014-02-28

**Authors:** Satu Kalliola, S Anna Pelkonen, Pekka L Malmberg, J Mika Mäkelä

**Affiliations:** 1 Skin and Allergy Hospital, Helsinki University Central Hospital, Helsinki, Finland

## Background

Airway hyperresponsiveness (AHR) is a key feature of asthma. AHR with asthma may begin already in infancy and can be assessed by direct and indirect bronchial challenges. Most young children are not able to perform maneuvers required for spirometry but can satisfactory perform impulse oscillometry (IOS) which needs minimal cooperation.

## Objective

To compare bronchial challenge tests by mannitol, methacholine and exercise with oscillometric technique in young children with obstructive symptoms.

## Methods

A total of 121 children (3.7-8.1 yr) were studied (31 with troublesome lung symptoms (TLS), 15 with bronchopulmonary dysplasia (BPD), 61 with history of early wheezing disorder and 14 healthy controls) to assess AHR by exercise test, mannitol and methacholine challenges with IOS. If the child used asthma control medication, it was stopped four weeks before the lung function tests. Tests were performed in separate days within two weeks period. AHR to exercise was defined as a ≥ 35 % increase in Rrs5. For mannitol and methacholine challenges, the dose causing an increase of 40 % in Rrs5 (PD40Rrs5) was calculated.

## Results

**Figure 1:**
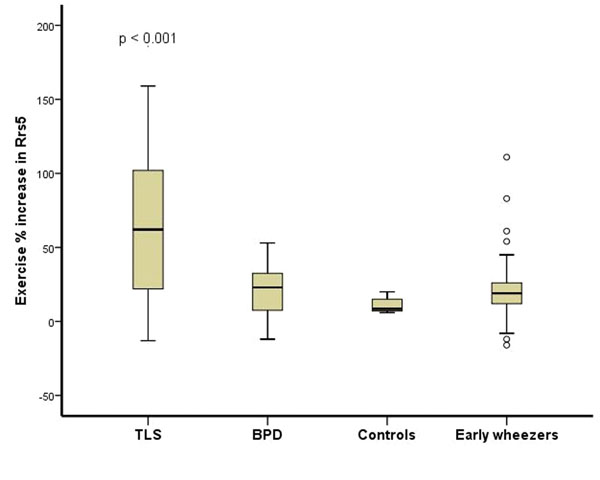


**Figure 2:**
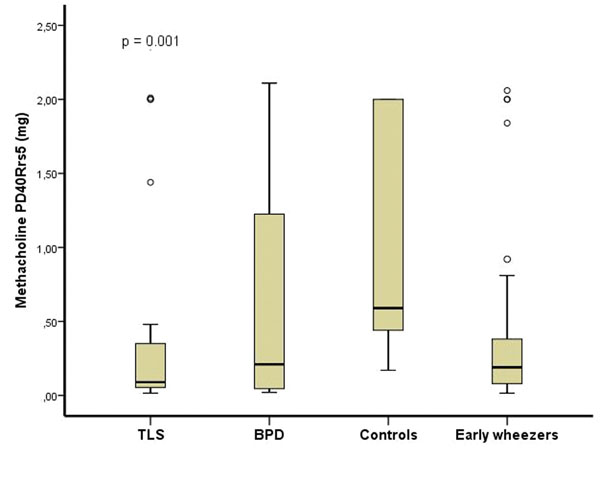


All 121 study children performed exercise and methacholine tests. Both tests distinguished well children with TLS from others (Figure 1 and 2). Mannitol test was satisfactory completed by 88 children. Ten children (11%) had positive mannitol challenge, but the test did not distinguish the study groups (p=0.209). The children with positive exercise challenges were more reactive to methacholine compared to children with negative exercise test (p=0.004). Atopy was found in 38 % of the study children.

## Conclusion

Mannitol challenge did not distinguish the study groups of young children with obstructive lung symptoms. Methacholine test is easy to perform with IOS, shows concurrent results with exercise challenge and may thereby offer a practical aid for evaluation of troublesome lung symptoms in young children.

